# The β-carboline Harmine improves the therapeutic benefit of anti-PD1 in melanoma by increasing the MHC-I-dependent antigen presentation

**DOI:** 10.3389/fimmu.2022.980704

**Published:** 2022-11-15

**Authors:** Muhammad Zaeem Noman, Irene Adelaide Bocci, Manale Karam, Kris Van Moer, Manon Bosseler, Akinchan Kumar, Guy Berchem, Christian Auclair, Bassam Janji

**Affiliations:** ^1^ Tumor Immunotherapy and Microenvironment (TIME) group, Department of Cancer Research, Luxembourg Institute of Health (LIH), Luxembourg City, Luxembourg; ^2^ AC Bioscience, Biopôle, Route de la Corniche 4, Epalinges, Switzerland; ^3^ AC Biotech, Villejuif Biopark, Villejuif, France; ^4^ Department of Hemato-Oncology, Centre Hospitalier du Luxembourg, Luxembourg City, Luxembourg

**Keywords:** MHC-I antigen presentation, harmine, DYRK1A, anti-PD1 Immunotherapy, T lymphocyte and NK cells infiltration, melanoma, inflammatory chemokines

## Abstract

Harmine is a dual-specificity tyrosine-regulated kinase 1A (DYRK1A) inhibitor that displays a number of biological and pharmacological properties. Also referred to as ACB1801 molecule, we have previously reported that harmine increases the presentation of major histocompatibility complex (MHC)-I-dependent antigen on melanoma cells. Here, we show that ACB1801 upregulates the mRNA expression of several proteins of the MHC-I such as Transporter Associated with antigen Processing TAP1 and 2, Tapasin and Lmp2 (hereafter referred to as MHC-I signature) in melanoma cells. Treatment of mice bearing melanoma B16-F10 with ACB1801 inhibits the growth and weight of tumors and induces a profound modification of the tumor immune landscape. Strikingly, combining ACB1801 with anti-PD1 significantly improves its therapeutic benefit in B16-F10 melanoma-bearing mice. These results suggest that, by increasing the MHC-I, ACB1801 can be combined with anti-PD1/PD-L1 therapy to improve the survival benefit in cancer patients displaying a defect in MHC-I expression. This is further supported by data showing that *i)* high expression levels of TAP1, Tapasin and Lmp2 was observed in melanoma patients that respond to anti-PD1; *ii)* the survival is significantly improved in melanoma patients who express high MHC-I signature relative to those expressing low MHC-I signature; and *iii)* high expression of MHC-I signature in melanoma patients was correlated with increased expression of CD8 and NK cell markers and overexpression of proinflammatory chemokines involved in the recruitment of CD8+ T cells.

## Introduction

Immune escape represents a major obstacle to successful cancer treatment based on immune checkpoint inhibitors (ICIs) (1). To escape CD8 T lymphocyte recognition, tumor cells lose their antigenicity through loss of immunogenic tumor antigens or defects in the antigen presentation machinery mediated by MHC-I (2). Moreover, malignant cells can gain additional aggressive properties by releasing factors that are responsible for the establishment of an immunosuppressive tumor microenvironment and the expression of immune checkpoint ligands (3).

MHC-I antigen presentation is the mechanism responsible for presenting “foreign” proteins on the surface of APC (antigen presenting cells) or cancer cells, thus allowing their recognition by CD8 T cells. Endogenously synthesized proteins are subjected to continuous degradation by the immunoproteasomes which is composed of several proteins, such the proteasome activator complexes 28α and β (PA28α and β ) and low-molecular-weight proteins 2 and 10 (LMP2 and LMP10). Such degradation process is needed for the generation of a majority of MHC-I-presented peptides. Some peptides produced by the immunoproteasomes (containing 9 to 13 residues) are transferred into the lumen of the endoplasmic reticulum (ER) by the Transporter Associated with antigen Processing (TAP) complex, which is composed of two different subunits (TAP1 and TAP2). The heavy and light β2-microglobulin (β2M) chains of MHC-I molecules are co-transported into the ER where they fold into the MHC-I heterodimer.

Upon transport into the lumen of the ER, peptides are in the vicinity of newly assembling MHC I molecules. The complex is stabilized through interactions with chaperones such as calreticulin. Additional component of this complex includes the peptide “editor” Tapasin which helps in maintaining peptide-empty MHC I molecules in the ER. Assisted by the peptide-editors Tapasin, MHC-I molecules can bind peptides displaying the right length and sequences. Stable peptide-MHC I complexes are finally released from the ER to be exposed to the plasma membrane and displayed to CD8 T cells by the exocytic pathway (reviewed in (4)).

Despite the success of anti-PD-1 therapy, many patients experience intrinsic or acquired resistance involving several non-mutually exclusive mechanisms (5). In addition to the low mutational burden, the most straightforward cause of the lack of responsiveness to anti-PD-1/PD-L1 is defects in the recognition of tumor cells by T cells, which can be related to the absence of tumor antigens or defects in the antigen presentation mechanism by MHC (6). Therefore, improving tumor antigen presentation by cancer cells is an attractive clinical approach to restore anti-tumor immunity and improve anti-PD-1 therapy.

The beta-carboline alkaloid harmine inhibits members of the dual-specificity tyrosine-regulated kinases (DYRK), including DYRK1A, DYRK1B, DYRK2, and DYRK4, with highest affinity for DYRK1A (7). In addition to its wide range of pharmacological activities, harmine displays anti-tumor properties by suppressing cell proliferation and inducing cell death in breast, lung, and ovarian cancers (8–11) and sensitizing pancreatic cancer cells to gemcitabine (12). Harmine plays a role in the actin cytoskeleton-dependent tumor reversion process (13), a key element in the formation of immunological synapses between T-cell receptors (TCRs) and MHC-I expressing tumor cells (14).

In this study we assessed the impact of the beta-carboline derivative ACB-1801 on the expression of proteins of the MHC-I and evaluate the functional significance on the improvement of anti-PD-1 therapy

Here, we report that, in B16-F10 melanoma cells, ACB1801 upregulates the expression of proteins involved in the MHC-I peptide-loading complex, such as transporter associated with antigen processing proteins 1 and 2 (TAP1 and TAP1), Tapasin and low-molecular-weight proteins 2 (Lmp2). The therapeutic value of ACB1801-dependent increase of TAP1, TAP2, Tapasin and Lmp2 is underscored by clinical data showing that high expression levels of these proteins was observed in melanoma patients that respond to anti-PD-1 and associated with an improved survival of melanoma patients. Using B16-F10 as melanoma mouse model, we show that ACB1801 treatment induces a profound modification of the tumor immune landscape and significantly improves the therapeutic benefit of anti-PD-1.

## Materials and methods

### Cells and reagents

B16-F10, GEMM, A375, and CT26 cell lines were purchased from ATCC and cultured as described in the data sheet of ATCC and a previous report (15). U87 and U251 cells were kindly provided by Dr. Anna Golebiewska (NorLux laboratory, LIH, Luxembourg) and cultured in Dulbecco’s modified Eagle’s medium-F12 (DMEM/F12, Gibco) supplemented with 10% fetal bovine serum (Gibco), 50-U/ml penicillin, and 250-μg/ml streptomycin. All cells were cultured in an incubator at 37°C with 5% CO2. All cell lines were frequently checked for whether they were free of mycoplasma using a Mycoalert kit (Lonza). ACB1801 was provided by AC Bioscience (Lausanne, Switzerland), mouse Tap1 monoclonal antibody (3D4), goat anti-mouse Phycoerythrin conjugated IgG secondary antibody and mouse IgG1 Isotype control (11711) were obtained from Novus, and MHC-class I H-2K^b^ antibody was obtained from Invitrogen.

### RNA extraction and SYBR Green real-time (RT)-qPCR

As reported previously (16), total RNA was extracted using TRIzol solution (Invitrogen) according to manufacturer’s instructions. 1 μg of total RNA was treated with DNase I and converted into cDNA using TaqMan Reverse Transcription Reagent (Applied Biosystems). The mRNA expression levels were quantified by the SYBR-GREEN qPCR method (Applied Biosystems). Relative expression was calculated using a comparative Ct method (2-ΔCt). The primer sequences are available upon request.

### *In vivo* study approval

Animal experiments were conducted according to the European Union guidelines. The *in vivo* experimentation protocols were approved by the LIH ethical committee, Animal Welfare Society, and Luxembourg Ministry of Agriculture, Viticulture and Rural Development (agreements n. LECR-2018-12).

### *In vivo* tumor growth and mouse treatments

C57BL/6 mice (7 weeks old) were purchased from Janvier and housed in pathogen-free conditions for one week before experiments. The mice were injected subcutaneously in the right flank with cell lines diluted in 100 µl of PBS. ACB1801 was administered to them with doses of 50, 20, and 10 mg/kg by oral gavage (per os) or 1 mg/kg by an i.p. route. Vehicle treatment was performed using methyl cellulose.

InVivoMab anti-mouse PD-1 (CD279) (BE0273) and InVivoMab rat IgG2a isotype control (BE0089) were purchased from BioXCell (Lebanon, USA), diluted in InVivoPure pH 7.0 Dilution Buffer (IP0070), and administered as indicated in the corresponding figures. Tumor volume (V) was measured using caliper every other day and estimated as follows: V (cm^3^) = ½ (Length × Width^2^). Mice were excluded if they did not develop tumors or developed tumors larger than the threshold defined in the approved experimentation protocols (volume > 2000 mm^3^), as previously reported (15).

### Tumor immune phenotyping and flow cytometry analysis

As previously reported (15), tumors were harvested, mechanically dissociated into fragments (<4 mm), and enzymatically digested using a mouse tumor dissociation kit (Miltenyi Biotec) for 45 min at 37°C. Single-cell suspensions were prepared, and red blood cells were lysed using Ammonium-Chloride-Potassium (ACK) lysis buffer (10-548E, Lonza). Live/Dead dye was used to select only live cells which were then counted using a Countess Automated Cell Counter (Invitrogen) and blocked for 30 minutes on ice with Fc block (TruStain fcX™ (anti-mouse CD16/32) Antibody 101320 Biolegend). Samples were stained for surface markers for lymphoid and myeloid immune populations. For FoxP3 and intracellular staining, True-Nuclear™ Transcription Factor Buffer Set 424401 Biolegend was used according to the manufacturer’s recommended protocol. CD45^+^ CD3^-^ NK1.1^+^ cells were defined as NK cells. Lymphocytes were defined as the CD3^+^ subpopulation of the CD45^+^ NK1.1^-^ gate. CD4+ and CD8^+^ T lymphocytes were derived from the CD3^+^ subpopulation. Tregs were subdivided from CD4^+^ T lymphocytes and defined as Foxp3^+^ and CD4^+^ Foxp3^-^ cells were considered as CD4^+^ T effector cells population. CD45^+^ CD11b^+^ cells were defined as a subset of live myeloid cells. DC were defined as the CD11c^+^ sub-population of the CD45^+^ CD11b^+^ subset. Polymorphonuclear MDSCs (PMN-MDSCs) were defined as the Ly6G^+^ Ly6C^low^ subpopulation of CD45^+^ CD11b^+^ subset. Total macrophages were defined as the F480^+^ subpopulation of the CD45^+^ CD11b^+^ subset. Inflammatory anti-tumoral macrophages (M1) were defined as F4/80^+^ CD206^-^, and protumoral macrophages (M2) were defined as F4/80^+^ CD206^+^ subpopulations of the F480^+^ CD45^+^ CD11b^+^ cells. The percentages of the different immune cell populations described above were calculated by reporting back to the total CD45^+^ live cells.

For flow cytometry, cells were harvested in 10 mM EDTA (Invitrogen). Surface staining was done at 4°C for 30 min using appropriate antibodies according to according to the manufacturer’s protocol. Dead cells were excluded using Live/Dead staining Kits (L34976; Thermo Fischer Scientific) or BD Via-Probe™ Cell Viability Solution (555815; Becton Dickinson). Samples were processed on a CytoFLEX flow cytometer and analyzed using CytExpert software.

The following antibodies were purchased from Biolegend: FITC anti-mouse CD45, Brilliant Violet 785 anti-mouse CD3, APC anti-mouse CD8a, APC/Fire 750 anti-mouse CD4, PE/Cy7 anti-mouse CD49b (pan-NK cells), PE/Cy7 anti-mouse NK-1.1 antibody, Brilliant Violet 605 anti-mouse CD69, PE/Cy5 anti-mouse CD25, Brilliant Violet 421 anti-mouse FOXP3, PE/Dazzle 594 anti-mouse CD279 (PD-1), Brilliant Violet 785 anti-mouse/human CD11b, APC anti-mouse F4/80, PE/Cy5 anti-mouse CD11c, PE/Cy7 anti-mouse Ly-6G, APC/Fire 750 anti-mouse Ly-6C, Brilliant Violet 605 anti-mouse CD206 (MMR), and Brilliant Violet 421 anti-mouse CD274 (B7-H1, PD-L1). A LIVE/DEAD Fixable Blue Dead Cell Stain Kit (ThermoFisher Scientific) was used for viability dying. For compensation controls, single dye stains were performed and the fluorescence spread was checked using Fluorescence Minus One (FMO) controls. The levels of non-specific binding was evaluated using isotype controls.

### Melanoma patient data mining

RNA expression reported as FPKM (Fragments Per Kilobase Million) values of anti-PD-1 treated melanoma patients from GEO (GSE78220) were retrieved and clinical data were downloaded from the corresponding published paper (17) for all the individual patients reported as responders or no responders to anti-PD-1. The FPKM value of MHC-I signature [TAP1, TAP2, TAPBP (Tapasin) and PSMB9 (Lmp2)] gene was compared between the two groups (Responders vs Non-responders). Mann Whitney U test was used to compute statistical significant difference using Graphpad Prism 8 software. Data from the TCGA skin cutaneous melanoma (SKCM) cohort (448 patients) were downloaded from cBioPortal (http://www.cbioportal.org/). IDs of patients displaying high and low TAP1, TAP2, TAPBP (Tapasin) and PSMB9 (Lmp2) mRNA expression (z-score relative to all samples) were extracted. Each patient’s vital status and survival values (overall survival and disease-specific survival) were downloaded from the TCGA database. In patients displaying high and low MHC-I signature, the log2 mRNA expression levels (batch normalized from Illumina HiSeq_RNASeqV2) of markers for NK (NCR1 and NCR3), CD8 (CD8A, CD8B, KLRG1), Cytotoxicity [Granzyme B (GZMB), Perforin (PER), TNF alpha (TNF) and Interferon gamma (IFNg)] were identified. The expression of inflammatory chemokines (CCL2, CCL4, CCL5, CCL19, CCL21, CXCL9, CXCL10, CXCL11, CXCL13, XCL2) was extracted from patients displaying low and high levels of MHC-I signature, NK markers and CD8 markers. The differential expression of genes of interest was found using GraphPad software. The median survival and the p-value were calculated using the log-rank (Mantel-Cox) test in GraphPad software.

### Statistical analysis

Statistical analyses were performed using GraphPad Prism 8. An unpaired two-tailed t-test was used to determine p-values between indicated groups. Results are represented as the mean ± standard error of the mean (SEM). A p-value < 0.05 was considered statistically significant (p ≤ 0.05 = *; p ≤ 0.01 or ≤ 0.05 = **; p ≤ 0.001 or ≤ 0.005 = ***; p > 0.05 = not significant, ns).

## Results

### ACB1801 increases the expression of several proteins of the MHC-I in various murine and human cancer cells

**Figure 1:**
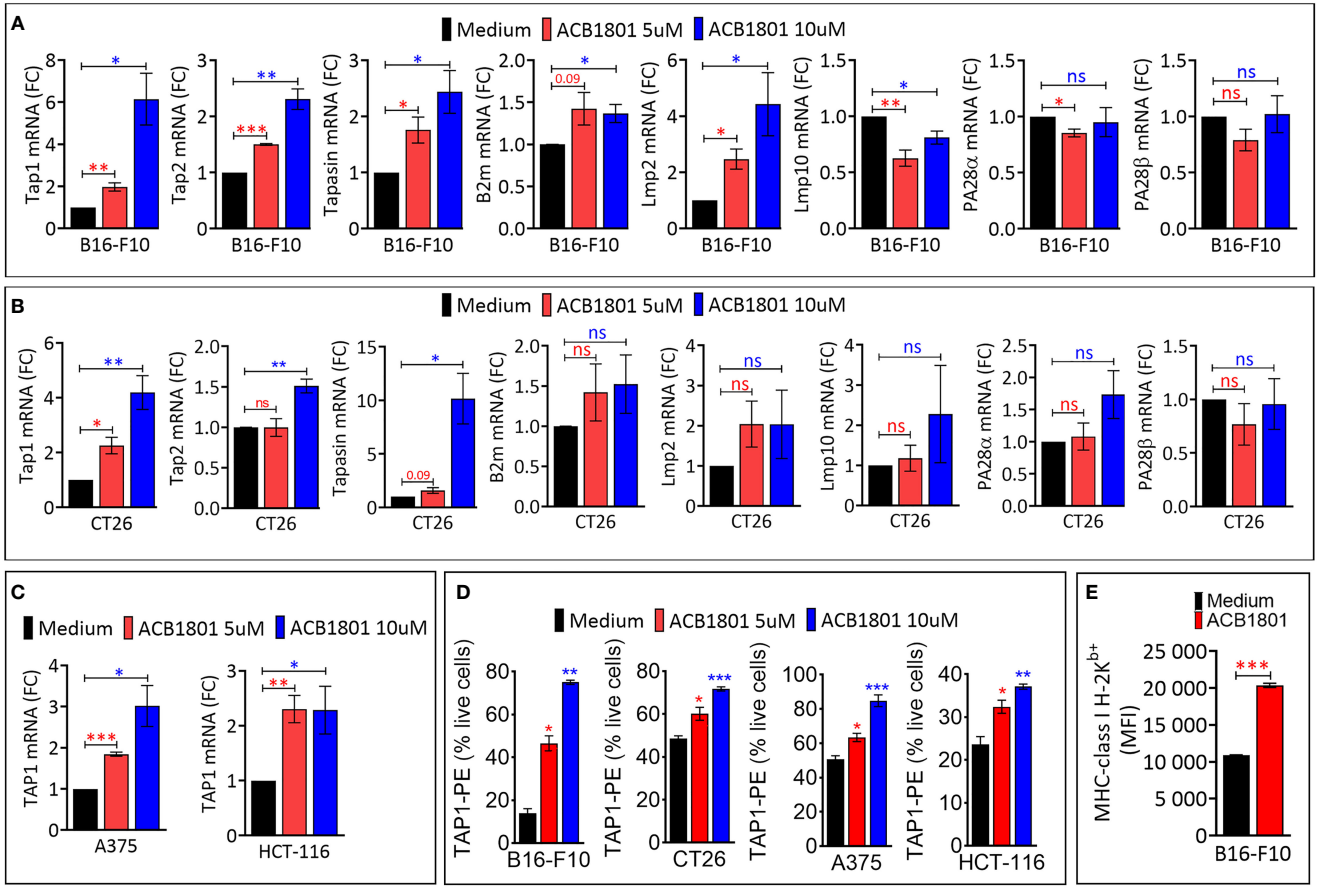
ACB1801 upregulates the expression of TAP1 in various murine and human tumor cells **(A, B)**. The mRNA expression of Tap1, Tap2, Tapasin, B2m, Lmp2, Lmp10, PA28α, PA28β, in mouse melanoma B16-F10 **(A)** and colorectal CT26 **(B)** cancer cells. **(C)** The mRNA expression of TAP1 in human melanoma A375 and colorectal HCT-116 cells. B16-F10, A375, CT26, and HCT-116 cells were treated for 24 h with culture medium (control) or with two concentrations of ACB1801 (5 and 10 uM). Results are reported as fold change (FC) relative to control cells treated with medium (black bars). **(D)** Flow cytometry analysis of the expression of TAP1 protein in B16-F10, CT26, A375, and HCT-116 cells treated for 24 h with culture medium (control) or two concentrations of ACB1801 (5 and 10 uM). Results are reported as % of positive cells relative to live cells. **(E)** Flow cytometry analysis of the expression of MHC-class I H-2K^b^ allotype on the cell surface of B16-F10 cells treated for 24 h with culture medium (control) or ACB1801 (5 uM). Results in **(A–E)** represent the averages of three independent experiments and are shown as mean ± SEM (error bars). Statistically significant differences were calculated relative to control conditions using an unpaired two-tailed student’s t-test (ns, not significant, * =p< 0.05, ** =p< 0.005, and ***=p<0.0005).

We assessed the impact of ACB1801 on the expression of antigen presentation genes (TAP1, TAP2, Tapasin, b2m, Lmp2, Lmp10, PA28α and PA26β) in murine B16-F10 melanoma. B16 cells are poorly immunogenic because they express low levels of MHC-I. This deficiency is attributed to the downregulation or loss in expression of multiple components of the MHC-I antigen-processing machinery (18). Cells treated with the culture medium alone were used as a control to evaluate the basal expression levels. We showed that ACB1801 upregulates the mRNA expression of TAP1, TAP2, Tapasin and Lmp2 genes involved in the antigen presentation in B16-F10 in a dose-dependent manner (**Figure 1A**).

The increased expression of TAP1, TAP2, Tapasin was also observed in colorectal CT26 cancer cells treated with 10 μM ACB-1801 (**Figure 1B**). Furthermore, the overexpression of TAP1, as a representative protein of the MHC-I, was detected in human melanoma cells A375, and colorectal HCT166 cells (**Figure 1C**) as well as in glioblastoma (U87 and U251) cells (**Supplementary Figure 1**). The increase of TAP1 mRNA by ACB1801 was translated into an increase of the protein expression of TAP1 in a dose-dependent manner (**Figure 1D**).

Initially named H−2, murine MHC-I comprises three gene loci: H−2K, H−2D, and H−2L. Several allotypes of these have been described, including H-2K^b^ (19). B16-F10 melanoma cells express low to undetectable levels of H-2K^b^ (20) which is critical for peptide-binding of murine MHC-I (21). We have previously reported that treatment of B16-F10 cells with ACB1801 increases H-2K^b^ bound OVA (SIINFEKL) peptide presentation by MHC-I (22). We believe that this could be related to an increase in the expression of the H-2K^b^ variant on the surface of B16-F10 cells following treatment with ACB1801, as shown in **Figure 1D**.

### ACB1801 inhibits B16-F10 tumor growth and improves the therapeutic benefit of anti-PD1

**Figure 2:**
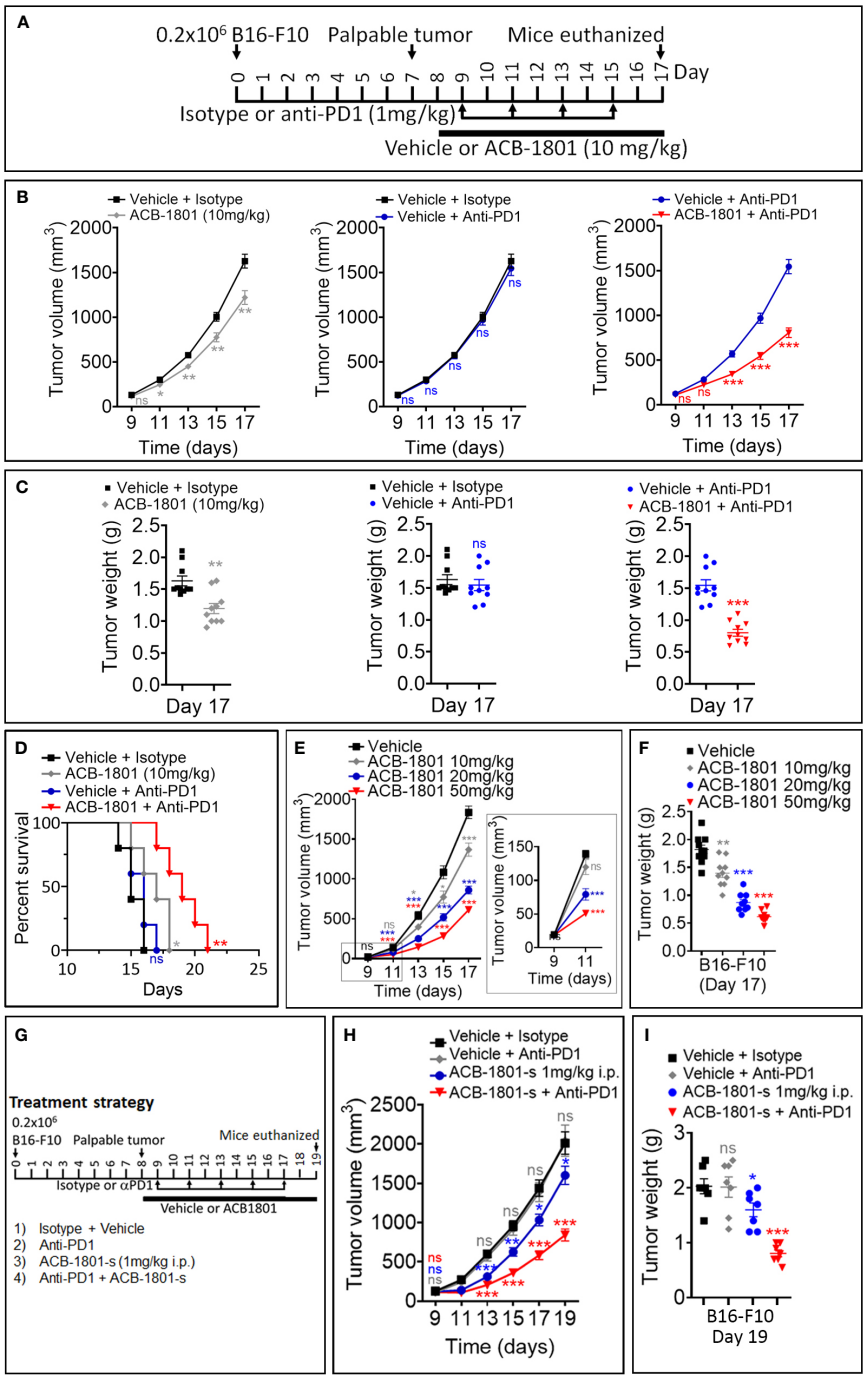
ACB1801 inhibits B16-F10 melanoma tumor growth and improves the therapeutic benefit of anti-PD-1. **(A)** Experimental schedule of B16-F10 melanoma treatment with mono and combination therapies of ACB1801 and/or anti-PD-1. B16-F10 cells (0.2 x 10^6^ cells) were injected subcutaneously in the right flank of C57BL/6 mice at day 0. Palpable tumors were observed at day 8. Treatment with ACB1801 (10 mg/kg) or vehicle was started at day 8 to day 17 and delivered daily per os. Treatment with anti-PD-1 (αPD-1, 1 mg/kg) was delivered i.p. at days 9, 11, 13, and 15. Mice were euthanized at day 17. **(B–D)** Tumor growth curves **(B)**, weight (g) at day 17 **(C)**, and mice survival **(D)** of B16-F10 melanoma in mice treated with vehicle and isotype (vehicle + isotype), ACB1801 (10 mg/kg) and isotype (ACB-1801 10 mg/kg), vehicle and anti-PD-1 (vehicle + anti-PD-1), or ACB1801 (10 mg/kg) and anti-PD-1 (ACB1801 + anti-PD-1). Results are reported as the average of 10 mice per group and shown as mean ± SEM (error bars). Statistically significant differences are calculated using an unpaired two-tailed student’s t-test (ns= not significant, * =p< 0.05, ** =p< 0.005, and ***=p<0.0005). Mice survival curves (5 mice per group) were generated from B16-F10 tumor-bearing mice. Lack of survival was defined as death or tumor size >1000 mm^3^. Mice survival percentage was determined using Graph Pad Prism, and p-values were calculated using the log-rank (Mantel-Cox) test (* = p ≤ 0.05, ** = p ≤ 0.01). **(E, F)** Tumor growth curves **(E)** and weight (g) at day 17 **(F)** of B16-F10 melanoma in mice treated with vehicle or ACB1801 at 10, 20, and 50 mg/kg. Results are reported as the average of 10 mice per group. Enlargement of the tumor growth at days 9 and 11 is shown in the right of panel **(E)** Results are shown as mean ± SEM (error bars). Statistically significant differences are calculated using an unpaired two-tailed student’s t-test (ns, not significant, and ***=p<0.0005). **(G–I)** Experimental schedule **(G)**, tumor growth **(H)**, and tumor weight **(I)** of B16-F10 melanoma treatment with mono and combination therapies of ACB1801 and/or anti-PD-1. B16-F10 cells (0.2 x 10^6^ cells) were injected subcutaneously in the right flank of C57BL/6 mice at day 0. Palpable tumors were observed at day 8. Treatment with ACB1801 (1 mg/kg) or vehicle was started at day 8 to day 17 and delivered daily by i.p. injection. Treatment with anti-PD-1 (anti-PD1, 1 mg/kg) was delivered i.p. at days 9, 11, 13, and 15. Mice were euthanized at day 17. Tumor growth curves **(H)** and weight in g at day 17 **(I)** of B16-F10 melanoma in mice treated with vehicle and isotype (vehicle + isotype), ACB1801 and isotype (ACB1801-s 1 mg/kg), vehicle and anti-PD-1 (anti-PD1), or ACB1801 and anti-PD-1 (ACB-1801-s + anti-PD1). Results are reported as the average of 7 mice per group as mean ± SEM (error bars). Statistically significant differences are calculated using an unpaired two-tailed student’s t-test (ns, not significant, * =p< 0.05, ** =p< 0.005, and ***=p<0.0005).

The efficacy of anti-PD-1 therapy relies on the effectiveness of neoantigen presentation by MHC-I on the surfaces of cancer cells (23). Melanoma patients displaying a low expression level of MHC-I are unlikely to benefit from anti-PD-1 (24). Based on these data, we assessed the impact of combining ACB1801 on the therapeutic benefit of anti-PD-1. We used a B16-F10 tumors since they are poorly immunogenic (18) and they do not respond to anti-PD-1 (15). The treatment schedule is shown in **Figure 2A**. Our results demonstrate that treatment with ACB1801 alone (10 mg/kg per os) decreased the tumor growth and weight of B16-F10 tumors and prologue the survival of tumor-bearing mice (**Figures 2B, C**, left panels, **D**). This effect is not restricted to B16-F10 tumors but is also observed in genetically engineered mouse melanoma (GEMM) tumors **(** **Supplementary Figure 2** **)** harboring genetic alterations (Braf^V600E/wt^ Pten^−/−^ Cdkn2^−/−^) found in human melanomas.

We also showed that anti-PD-1 monotherapy had no effect on B16-F10 tumor growth, tumor weight and mice survival, as expected (**Figures 2B, C**, middle panels, and **D**). However, combining ACB1801 with anti-PD-1 remarkably improved the therapeutic benefit compared to anti-PD-L1 monotherapy (**Figures 2B, C**, right panels and **D**). Our results depicted in **Figures 2E, F** further indicate that ACB-1801 inhibits the growth of B16-F10 tumors in a doses dependent manner.

We next assessed whether lower doses of ABC1801 are still able to inhibit B16-F10 melanoma tumor growth and improve the therapeutic benefit of anti-PD-1. We found that ACB1801 at 1 mg/kg i.p. significantly improves the therapeutic benefit of anti-PD-1 (**Figures 2G–I**). Our results provide strong evidence that treatment with ACB1801 makes non-responder B16-F10 tumors strong responders to anti-PD1.

### ACB1801 modifies the immune landscape of B16-10 tumors and enhances the infiltration of various anti-tumor immune effector cells

We have previously reported that ACB1801 treatment had no effect on B16-F10 tumor-bearing immunodeficient NOD scid gamma mice (NSG) lacking mature B, T, and NK cells (22). These results indicate that ACB1801-dependent inhibition of B16-F10 melanoma tumor growth involves the immune system. To evaluate whether ACB-1801 impacts the infiltration of immune cells into the tumor microenvironment, we performed a comprehensive analysis of the immune landscape of ACB1801-treated tumors using the gating strategies for lymphoid and myeloid immune phenotyping, which we have defined previously (15).

**Figure 3:**
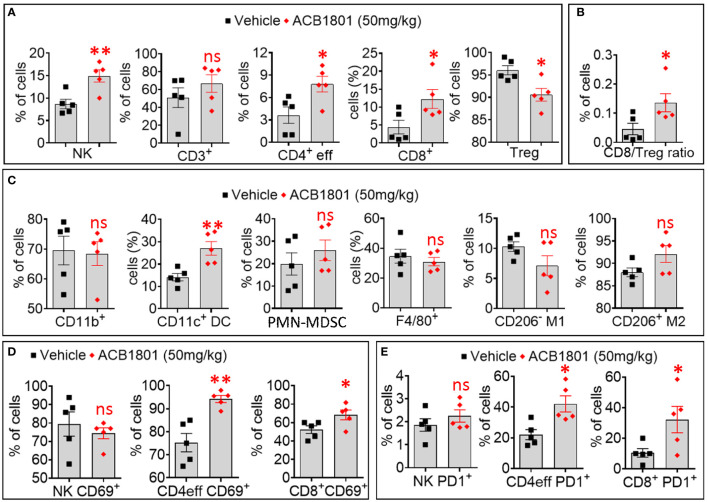
Treatment of B16-F10 tumor-bearing mice with ACB-1801 increases the infiltration of cytotoxic immune cells into the tumor microenvironment. **(A)** Flow cytometry quantification of the percent (%) live natural killer (NK) cells, CD3+, CD4+ effector T cells, CD8+ T cells, and regulatory T lymphocytes (Treg) infiltrating B16-F10 tumors treated treated with vehicle or 50 mg/kg of ACB-1801. **(B)** The ratio of CD8/Treg reported as percent (%) of cells infiltrating B16-F10 tumors treated as described in **(A)**. **(C)** Flow cytometry quantification of the percent (%) of live myeloid cells (CD11b+), dendritic cells (DC), polymorphonuclear myeloid derived suppressor cells (PMN-MDSC), total macrophages (F4/80), M1 macrophages (CD206^-^ M1), and M2 macrophages (CD206^+^ M2) infiltrating B16-F10 tumors treated as described in **(A)**. **(D, E)** Flow cytometry quantification of the percent (%) of live CD69^+^ **(D)**, PD-1^+^ **(E)** NK cells, CD4+ effector T cells, and CD8+ T cells infiltrating B16-F10 tumors treated as described in **(A)**. All quantifications were performed on well-established tumors harvested at day 17. The immune cell populations were gated and quantified in live CD45+ cells. Each dot represents one tumor. Data are reported as the average of 5 mice per group as mean ± SEM (error bars). Statistically significant differences are calculated in comparison to vehicle-treated tumors using an unpaired two-tailed student’s t-test (ns, not significant, * =p< 0.05, and ** =p< 0.005).

We showed a significant increase in the infiltration of NK cells, CD4 T effector (eff) cells, and CD8^+^ T cells. This was associated with a significant decrease in the infiltration of immunosuppressive Treg cells in ACB1801-treated B16-F10 tumors compared to vehicle-treated controls (**Figures 3A**). We therefore found that the ratio CD8/Treg was increase in ACB-1801-treated tumors compared to controls (**Figures 3B**). By analyzing the infiltration of myeloid immune cells, we showed that there is no significant difference was observed in the infiltration of total (CD11b^+^) myeloid cells, total (F4/80^+^) macrophages, M1 (CD206^-^) macrophages, M2 (CD206^+^), and polymorphonuclear myeloid derived suppressor cells (PMN-MDSCs), but a significant increase was detected in the infiltration of CD11c^+^ dendritic cells (DCs) in ACB1801-treated B16-F10 tumors compared to vehicle-treated controls (**Figure 3C**). To evaluate the functional status NK cells, CD4 T effector (eff) cells, and CD8^+^ T cells infiltrating ACB-1801-treated tumors, we evaluate the expression of the activation marker CD69 and the early exhaustion marker PD-1. Our data showed a significant increase in the expression of CD69 and PD-1 markers on CD4 T effector (eff) cells and CD8^+^ T cells, but not on NK cells, infiltrating ACB1801-treated B16-F10 tumors (**Figures 3D, E**).

### High expression level of MHC-I signature is associated with improved survival benefit, overexpression of CD8 and NK markers as well as high expression of chemokines associated with CD8+ T cell recruitment in melanoma patients

Based on our *in vitro* data, we have defined TAP1, TAP2, TAPASIN (TAPBP) and LMP2 as an MHC-I signature which is regulated by ACB-1801.

**Figure 4:**
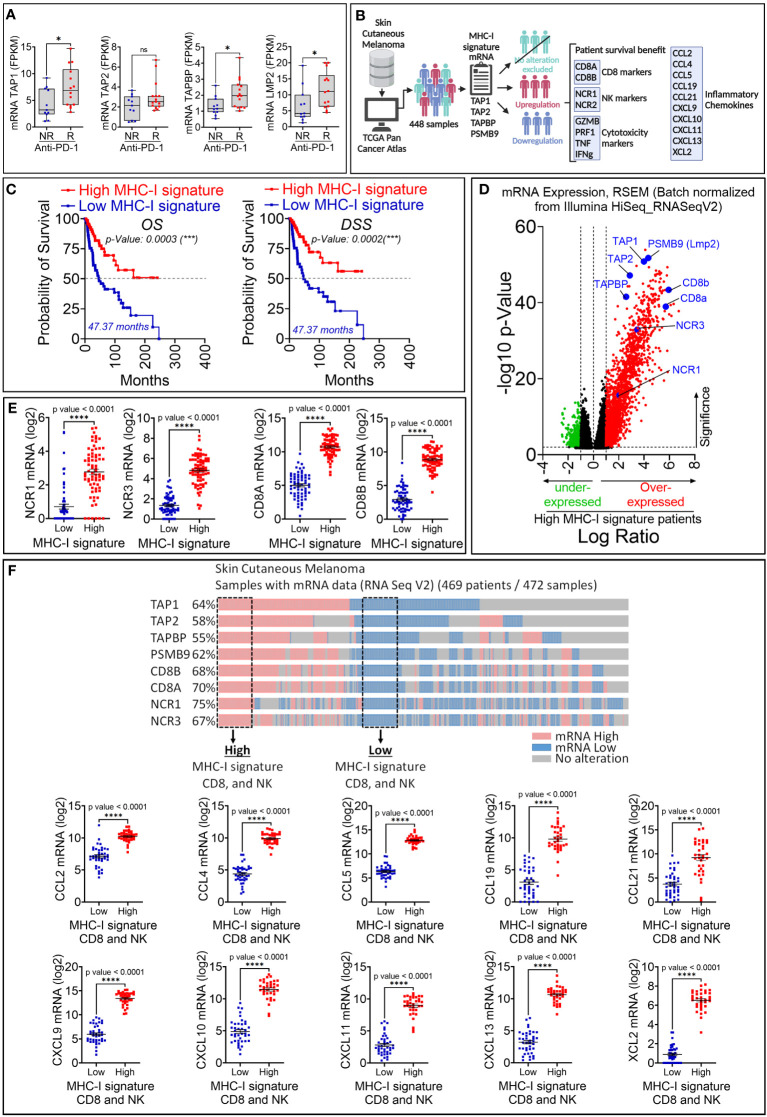
Retrospective analysis of the therapeutic value of MHC-I signature (TAP1, TAP2, TAPBP and LMP2) upregulation in melanoma patient cohorts. **(A)** The expression of MHC-I signature (TAP1, TAP2, TAPBP and LMP2) reported as FPKM in metastatic melanoma patients who are not responsive (NR) or responsive **(R)** to anti-PD-1. Statistically significant difference was determined using Mann Whitney U test in Graphpad Prism 9 software. **(B)** Workflow used for analyzing melanoma patient data in TCGA database. **(C)** Kaplan-Meier overall survival (OS, left panels) and disease-specific survival (DSS, right panels) curves of melanoma patients expressing high and low mRNA of MHC-I signature. Patients displaying high MHC-I signature have significantly improved OS and DSS compared to those with low MHC-I signature. The p-value of each curve was determined using the log-rank (Mantel-Cox) test. **(D)** Volcano plot of differentially expressed genes in melanoma patients with high and low mRNA expression of MHC-I signature. Scattered points represent genes. The x-axis shows the log2 fold change for the ratio of high compared to low expression of MHC-I signature. The y-axis shows significance by -log10 transformed p-value value. Red dots in the right of the dashed vertical line at +1 value represent genes that are significantly over-expressed in patients with high MHC-I signature. Green dots on left of the dashed vertical line at -1 value represent genes that are significantly under-expressed in in patients with high MHC-I signature. A gene is considered significantly differentially expressed if |log(FC)| ≥ 0.1 and |log10 (p-value)| ≤ 0.01. TAP1, TAP2, TAPBP, PSMB9, CD8 markers (CD8A and CD8B) and NK (NCR1 and NCR2) are shown in blue. **(E)** The mRNA expression of NK markers (NCR1 and NCR3) and CD8 markers (CD8A and CD8B) reported as log2 in melanoma patients displaying high and low MHC-I signature. Results are shown as mean ± SEM (error bars). Statistically significant differences of high MHC-I signature are calculated compared to patients with low MHC-I signature using an unpaired two-tailed student’s t-test (**** = p<0.0001). **(F)** *Upper panel:* Strategy used to extract melanoma patient data in TCGA database expressing low and high TAP1, TAP2, Tapasin (TAPBP), Lmp2 (PSMB9), CD8A, CD8A and NCR1 and NCR3 genes. Doted boxes define patients that we have considered to assess the expression of CCL2, CCL4, CCL5, CCL19, CCL21, CXCL9, CXCL10, CXCL11, CXCL13 and XCL2 chemokines. *Lower panel:* The mRNA expression of CCL2, CCL4, CCL5, CCL19, CCL21, CXCL9, CXCL10, CXCL11, CXCL13 and XCL2 in patients defined in the upper panel. The differential expression of genes of interest was defined using GraphPad software. Results are represented as the mean ± standard error of the mean (SEM). Statistically significant differences are calculated using an unpaired two-tailed student’s t-test (**** = p<0.0001).

To evaluate the therapeutic value of the MHC-I signature overexpression, we used clinical and RNA-seq data of patients treated with either pembrolizumab or nivolumab as the anti-PD-1 therapy for their metastatic melanoma (17). We showed that melanoma patients who were responsive to anti-PD-1 expressed significantly higher levels of TAP1, TAPASIN, LMP2, but not TAP2, compared to those who were not responsive to this therapy (**Figure 4A**). We next assessed the survival and expression of NK cell markers (NCR1 and NCR3) and CD8 T cell markers (CD8A and CD8B) in 448 patients with skin cutaneous melanoma reported in the TCGA database. The data mining workflow is shown in **Figure 4B**. Our results show that the overall survival (OS) and disease specific survival (DSS) are significantly higher in melanoma patients expressing high MHC-I signature than those expressing low MHC-I signature (**Figure 4C**). We also found that improved survival in patients expressing high MHC-I signature is associated with higher expression of NK and CD8 T cell markers (**Figures 4D, E**). Furthermore, we showed that the expression of the cytotoxic markers GZMB, PRF1, TNF, IFNg are significantly increased in patients displaying high MHC-I signature compared to those having low MHC-I signature (**Supplementary Figure 3**).

Our data suggest that melanoma patients expressing high MHC-I signature are more infiltrated by CD8 T cells compared to those expressing low MHC-I signature. We believe that the infiltration of CD8 T cells and NK cells is presumably due to the over-expression of chemokines involved in driving these cells in the tumor microenvironment. This assumption was supported by our data in **Figure 4F** showing high expression of the chemokine signature involved in the CD8 T cell recruitment (25) in patients displaying high MHC-I signature, CD8 and NK cells compared to those having low MHC-I signature, CD8 and NK cells. Interestingly, among patients with high MHC-I signature, 65% of them express high levels of effector CD8 T-cell markers (CD8a^+^ CD8b^+^ KLRG1^+^), while 50% of them express high levels of Treg markers (CD4^+^ Foxp3^+^ ISG20^+^) (**Supplementary Figure 4A**). Moreover, we also found that 46% of patients with high MHC-I signature express increased M2 markers ADGRE1^+^ (Adhesion G Protein-Coupled Receptor E1) and MRC1^+^ (Mannose Receptor C-Type 1) which are almost all positive for CD274 (PD-L1), but not for ARG1 (Arginase 1) (**Supplementary Figure 4B**). These results suggest that, in addition to cytotoxic effector cells, immunosuppressive cells and M2 macrophages could also be present in the tumor microenvironment of tumors expressing high MHC-I signature. Nevertheless, the function of immunosuppressive cells needs to be deeply investigated under these conditions.

Information about the TCGA melanoma patients is shown in **Supplementary Tables 1**–**6**. Collectively, these data highlight the therapeutic value of increasing the expression levels of TAP1, TAP2, TAPBP and PSMB9 in melanoma.

## Discussion

Numerous studies are currently ongoing to understand the resistance mechanisms to immune checkpoint blockades and explore novel combinatorial approaches. We have shown that the β-carboline derivative ACB1801 potentiates the therapeutic benefit of anti-PD-1 in a B16-F10 melanoma mouse model, reported to resist to anti-PD-1 therapy (26, 27) and to express low levels of MHC-I (18). B16-F10 is, therefore, an appropriate mouse model for investigating the properties of molecules regulating MHC-I and assessing strategies to overcome the resistance to anti-PD-1/PD-L1.

Our *in vitro* results showed that ACB1801 increases the expression of several proteins of the MHC-I such as TAP1, TAP2, TAPBP, and the low-molecular-weight protein 2 (LMP2). Therefore, it is tempting to speculate that the effect of ACB-1801 on the increase of the antigen presentation in B16-F10 cells, previously reported by us (22), could be the result of the upregulation of several proteins involved in the MHC-I.

The precise mechanism by which ACB-1801 increases the antigen presentation to MHC-I is still not fully understood. However, we believe that the mode of action of ACB-1801 relies on its ability to remodel the actin cytoskeleton through inhibiting DYRK1A. DYRK1A is a negative regulator of the actin-related protein 2/3 (Arp2/3), which is involved in the actin polymerization process through Wiskott–Aldrich syndrome protein (WASP) phosphorylation (7, 13, 28). Therefore, we argue that inhibiting DYRK1A by ACB1801 would enhance the reorganization of the actin cytoskeleton, a prerequisite process for TCR/MHC immune synapse stabilization between T cells and APC (29–31).

Our *in vivo* data showed that, even without combination with anti-PD-1, monotherapy with ACB-1801 alone significantly inhibits the growth of B16-F10 tumors. Consistence with these data, it cannot ruled out that, through remodeling the actin cytoskeleton, ACB-1801 induces a reversion of the aggressive phenotype of the B16-F10 cells, thereby making them more prone to immune recognition and killing. Indeed, accumulating evidence suggests that tumor reversion refers to a process where cancer cell lose their malignant phenotype (also termed bad or tumor escape phenotype) and gain a good phenotype (also called tumor rejected phenotype) due to extensive genetic reprogramming (32, 33). Tumor cells with bad phenotype are derived from established tumors after escaping T-cell-mediated immune surveillance (34). From these evidences, we suspect that ACB-1801 increases tumor antigen presentation most likely through its potent property to inhibit DYRK1A and revert tumor malignant phenotype. Although a direct experimental evidence of such a mechanism is still needed, this concept is supported by evidences showing that: i) several kinases negatively regulate MHC-I expression and antigen presentation machinery in multiple cancers (35); and ii) one of the major characteristics of the malignant phenotype is the impairment of tumor antigen presentation due to genetic aberrations that provide growth and survival benefit to tumors (36). Therefore, the reversion of the malignant phenotype has been proposed to result in the unmasking of tumor cells, which would mainly occur through the rescue of tumor antigen presentation (4). Nevertheless, it is unlikely that the anti-tumor effect of ACB1801 results from exclusive action on tumor cells.

**Figure 5:**
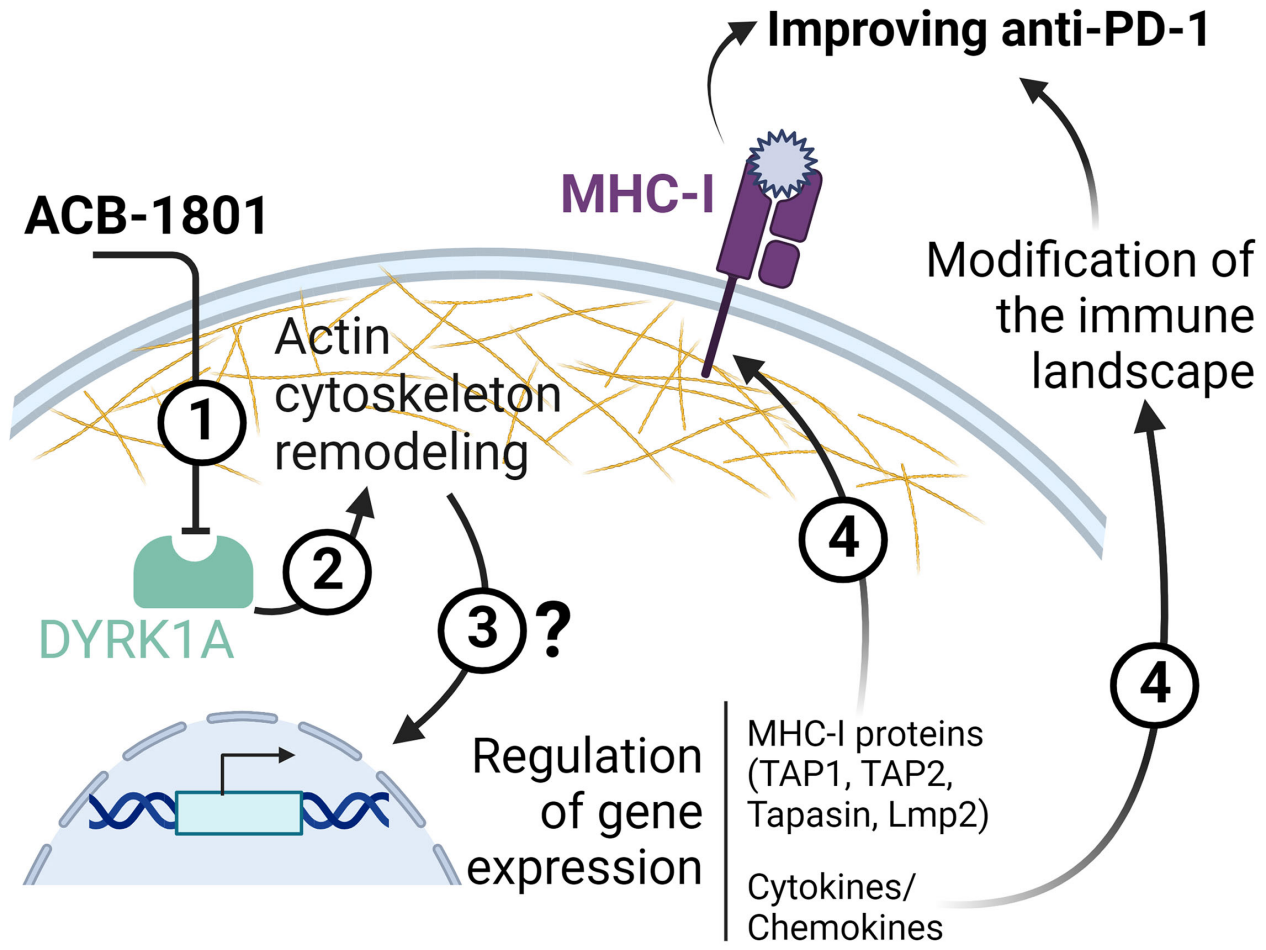
Schematic representation of the proposed role of ACB-1801. By inhibiting DYRK1A (1), ACB-1801 induces actin cytoskeleton remodeling (2) and phenotypic switch of cancer cells. Such phenotypic switch can regulate the expression of several genes (3) of the MHC-I and various cytokines and chemokines by mechanism(s) which are not fully understood. The regulation of several genes of MHC-I (TAP1, TAP2, Tapasin and Lmp2) leads to an overexpression of MHC-I (4). Whereas, the regulation of cytokine/chemokines genes induces a modification of the tumor immune landscape (4).

In addition to increasing the tumor antigen presentation, ACB1801 induces a deep modification of the immune landscape of B16-F10 tumors characterized by an increase of NK, CD4, and CD8 T cells and decrease of Tregs infiltration in the tumor microenvironment. Although the mechanism(s) underlying the tumor immune landscape modification by ACB1801 is still under investigation, we cannot exclude that one of these mechanisms relies on the regulation of the cytokine/chemokine repertoire in tumor cells, which are subjected to the phenotypic reversion. This statement is supported by previous studies showing that tumor cells undergoing phenotypic switch can regulate the release of cytokine/chemokine repertoire, thereby modifying the tumor immune landscape (37, 38). Schematic representation of the proposed role of ACB-1801 is provided in **Figure 5**.

Soluble factors released in the tumor microenvironment may act *via* an autocrine mechanism on tumor cells themselves or by a paracrine mechanism on other cells present in the tumor microenvironment, including immune cells. Therefore, it is tempting to speculate that the increased expression of CD69 and PD-1 on both CD4 effectors and CD8 T cells resulted from yet undefined soluble factors released by tumor cells. It should be highlighted that the overexpression of PD-1 on tumor-specific T cells should not only predict the exhaustion status, but can also be considered as a marker of activated tumor-reactive T cells (39, 40).

Nevertheless, further investigations must be carried out to profile the secretome of tumor cells treated with ACB1801 and understand its impact on the anti-tumor activity of different immune cells infiltrating treated tumors. In line with this work, previous study suggested that Harmine enhances the differentiation of Treg cells and strongly inhibits Th17 cell differentiation with minimal impact on Th1 responses *in vitro* (41). Based on our preclinical data showing an increased infiltration of cytotoxic immune cells in the tumor microenvironment of ACB-1801-treated tumors, which are further supported by clinical results, our results provides a framework for rational combination immunotherapy development of ACB-1801 and anti-PD-1.

The relevance of our study is underscored by clinical data showing that about 50% of cancer patients displayed an abnormal antigen presentation. Therefore, combining ACB1801 could substantially increase the number of cancer patients that would benefit from the impressive therapeutic value of ICI. Overall, our data can contribute to the emergence of a new wave of combination immunotherapy that would provide durable clinical outcomes and create support for immunotherapy.

## Data availability statement

Patient data that support the findings of this study were derived from the following resources available in the public domain: https://www.cbioportal.org/. Further inquiries can be directed to the corresponding author.

## Ethics statement

Animal experiments were conducted according to the European Union guidelines. The in vivo experimentation protocols were approved by the Luxembourg Institute of Health ethical committee, Animal Welfare Society, and Luxembourg Ministry of Agriculture, Viticulture and Rural Development (agreements n. LECR-2018-12).

## Author contributions

Study concept and experimental design: MZN, CA, and BJ; conducting experiments: MZN, KVM, IAB and MB; data acquisition: MZN, KVM, MB and AK; data analysis and interpretation: MZN, KVM, MB, AK, and BJ; study supervision: CA, GB, MK, GB, and BJ; writing the manuscript: BJ, CA, and MK. All authors contributed to the article and approved the submitted version.

## Funding

This work was supported by grants from the Luxembourg National Research Fund (CORE-C18/BM/12670304/COMBATIC; BRIDGES2020/BM/15412275/SMART-COMBO; BRIDGES2021/BM/16358198/TRICK-ALDH and INTER/EUROSTARS21/16896480/C2I), FNRS-Televie (7.4560.21-INCITE21; 7.4559.21-IMPACT21 and 7.4579.20-CD73), Fondation Recherche Cancer et Sang, Luxembourg (INCOM BIOM), Kriibskrank Kanner Foundation; Luxembourg (Neuroimmunotherapy II-2019); Action LION-S Vaincre le Cancer Luxembourg (AUTOKIR-2019, COMBO-KD2020 and AB-2020); Roche Pharma (2020) and Stiftelsen Cancera (2022).

## Conflict of interest

MK and CA are employees at AC Biotech and AC Bioscience, respectively. The remaining authors declare that the research was conducted in the absence of any commercial or financial relationships that could be construed as a potential conflict of interest.

## Publisher’s note

All claims expressed in this article are solely those of the authors and do not necessarily represent those of their affiliated organizations, or those of the publisher, the editors and the reviewers. Any product that may be evaluated in this article, or claim that may be made by its manufacturer, is not guaranteed or endorsed by the publisher.

## References

[1] Kim JM Chen DS . Immune escape to pd-L1/Pd-1 blockade: Seven steps to success (or failure). Ann Oncol (2016) 27(8):1492–504. doi: 10.1093/annonc/mdw217 27207108

[2] Beatty GL Gladney WL . Immune escape mechanisms as a guide for cancer immunotherapy. Clin Cancer Res (2015) 21(4):687–92. doi: 10.1158/1078-0432.CCR-14-1860 PMC4334715 25501578

[3] Labani-Motlagh A Ashja-Mahdavi M Loskog A . The tumor microenvironment: A milieu hindering and obstructing antitumor immune responses. Front Immunol (2020) 11:940. doi: 10.3389/fimmu.2020.00940 32499786 PMC7243284

[4] Dhatchinamoorthy K Colbert JD Rock KL . Cancer immune evasion through loss of mhc class I antigen presentation. Front Immunol (2021) 12:636568. doi: 10.3389/fimmu.2021.636568 33767702 PMC7986854

[5] Sharma P Hu-Lieskovan S Wargo JA Ribas A . Primary, adaptive, and acquired resistance to cancer immunotherapy. Cell (2017) 168(4):707–23. doi: 10.1016/j.cell.2017.01.017 PMC5391692 28187290

[6] Gubin MM Zhang X Schuster H Caron E Ward JP Noguchi T . Checkpoint blockade cancer immunotherapy targets tumour-specific mutant antigens. Nature (2014) 515(7528):577–81. doi: 10.1038/nature13988 PMC4279952 25428507

[7] Ogawa Y Nonaka Y Goto T Ohnishi E Hiramatsu T Kii I . Development of a novel selective inhibitor of the down syndrome-related kinase Dyrk1a. Nat Commun (2010) 1:86. doi: 10.1038/ncomms1090 20981014

[8] Yochum ZA Cades J Wang H Chatterjee S Simons BW O'Brien JP . Targeting the emt transcription factor Twist1 overcomes resistance to egfr inhibitors in egfr-mutant non-Small-Cell lung cancer. Oncogene (2019) 38(5):656–70. doi: 10.1038/s41388-018-0482-y PMC6358506 30171258

[9] Li YL Ding K Hu X Wu LW Zhou DM Rao MJ . Dyrk1a inhibition suppresses Stat3/Egfr/Met signalling and sensitizes egfr wild-type nsclc cells to Azd9291. J Cell Mol Med (2019) 23(11):7427–37. doi: 10.1111/jcmm.14609 PMC6815810 31454149

[10] Ding Y He J Huang J Yu T Shi X Zhang T . Harmine induces anticancer activity in breast cancer cells *Via* targeting taz. Int J Oncol (2019) 54(6):1995–2004. doi: 10.3892/ijo.2019.4777 31081045 PMC6521938

[11] Gao J Zhu H Wan H Zou X Ma X Gao G . Harmine suppresses the proliferation and migration of human ovarian cancer cells through inhibiting Erk/Creb pathway. Oncol Rep (2017) 38(5):2927–34. doi: 10.3892/or.2017.5952 28901502

[12] Wu LW Zhang JK Rao M Zhang ZY Zhu HJ Zhang C . Harmine suppresses the proliferation of pancreatic cancer cells and sensitizes pancreatic cancer to gemcitabine treatment. Onco Targets Ther (2019) 12:4585–93. doi: 10.2147/OTT.S205097 PMC6580126 31354292

[13] Le Moigne R Subra F Karam M Auclair C . The beta-carboline harmine induces actin dynamic remodeling and abrogates the malignant phenotype in tumorigenic cells. Cells (2020) 9(5):1168. doi: 10.3390/cells9051168 32397195 PMC7290983

[14] Roy NH Burkhardt JK . The actin cytoskeleton: A mechanical intermediate for signal integration at the immunological synapse. Front Cell Dev Biol (2018) 6:116. doi: 10.3389/fcell.2018.00116 30283780 PMC6156151

[15] Noman MZ Parpal S Van Moer K Xiao M Yu Y Viklund J . Inhibition of Vps34 reprograms cold into hot inflamed tumors and improves anti-Pd-1/Pd-L1 immunotherapy. Sci Adv (2020) 6(18):eaax7881. doi: 10.1126/sciadv.aax7881 32494661 PMC7190323

[16] Berchem G Noman MZ Bosseler M Paggetti J Baconnais S Le Cam E . Hypoxic tumor-derived microvesicles negatively regulate nk cell function by a mechanism involving tgf-beta and Mir23a transfer. Oncoimmunology (2016) 5(4):e1062968. doi: 10.1080/2162402X.2015.1062968 27141372 PMC4839360

[17] Hugo W Zaretsky JM Sun L Song C Moreno BH Hu-Lieskovan S . Genomic and transcriptomic features of response to anti-Pd-1 therapy in metastatic melanoma. Cell (2016) 165(1):35–44. doi: 10.1016/j.cell.2016.02.065 26997480 PMC4808437

[18] Seliger B Wollscheid U Momburg F Blankenstein T Huber C . Characterization of the major histocompatibility complex class I deficiencies in B16 melanoma cells. Cancer Res (2001) 61(3):1095–9. 11221838

[19] Pease LR Schulze DH Pfaffenbach GM Nathenson SG . Spontaneous h-2 mutants provide evidence that a copy mechanism analogous to gene conversion generates polymorphism in the major histocompatibility complex. Proc Natl Acad Sci U.S.A. (1983) 80(1):242–6. doi: 10.1073/pnas.80.1.242 PMC393348 6571997

[20] Calorini L Marozzi A Byers HR Waneck GL Lee KW Isselbacher KJ . Expression of a transfected h-2kb gene in B16 cells correlates with suppression of liver metastases in triple immunodeficient mice. Cancer Res (1992) 52(14):4036–41. 1617680

[21] Molano A Erdjument-Bromage H Fremont DH Messaoudi I Tempst P Nikolic-Zugic J . Peptide selection by an mhc h-2kb class I molecule devoid of the central anchor ("C") pocket. J Immunol (1998) 160(6):2815–23. 9510184

[22] Schlapfer A Auclair C Janji B Karam M Zaeem Noman M . The 'Holy grail' in immuno-oncology: Ac bioscience sa is aiming to potentiate anti-Pd-1 therapy efficacy through tumor cell conditioning strategy. Chimia (Aarau) (2020) 74(10):771–5. doi: 10.2533/chimia.2020.771 33115558

[23] Ribas A Wolchok JD . Cancer immunotherapy using checkpoint blockade. Science (2018) 359(6382):1350–5. doi: 10.1126/science.aar4060 PMC7391259 29567705

[24] Shklovskaya E Lee JH Lim SY Stewart A Pedersen B Ferguson P . Tumor mhc expression guides first-line immunotherapy selection in melanoma. Cancers (Basel) (2020) 12(11):3374. doi: 10.3390/cancers12113374 33202676 PMC7696726

[25] Harlin H Meng Y Peterson AC Zha Y Tretiakova M Slingluff C . Chemokine expression in melanoma metastases associated with Cd8+ T-cell recruitment. Cancer Res (2009) 69(7):3077–85. doi: 10.1158/0008-5472.CAN-08-2281 PMC3886718 19293190

[26] Wculek SK Amores-Iniesta J Conde-Garrosa R Khouili SC Melero I Sancho D . Effective cancer immunotherapy by natural mouse conventional type-1 dendritic cells bearing dead tumor antigen. J Immunother Cancer (2019) 7(1):100. doi: 10.1186/s40425-019-0565-5 30961656 PMC6454603

[27] Ueha S Yokochi S Ishiwata Y Ogiwara H Chand K Nakajima T . Robust antitumor effects of combined anti-Cd4-Depleting antibody and anti-Pd-1/Pd-L1 immune checkpoint antibody treatment in mice. Cancer Immunol Res (2015) 3(6):631–40. doi: 10.1158/2326-6066.CIR-14-0190 25711759

[28] Park J Sung JY Park J Song WJ Chang S Chung KC . Dyrk1a negatively regulates the actin cytoskeleton through threonine phosphorylation of n-wasp. J Cell Sci (2012) 125(Pt 1):67–80. doi: 10.1242/jcs.086124 22250195

[29] Calvo V Izquierdo M . Role of actin cytoskeleton reorganization in polarized secretory traffic at the immunological synapse. Front Cell Dev Biol (2021) 9:629097. doi: 10.3389/fcell.2021.629097 33614660 PMC7890359

[30] Dustin ML Cooper JA . The immunological synapse and the actin cytoskeleton: Molecular hardware for T cell signaling. Nat Immunol (2000) 1(1):23–9. doi: 10.1038/76877 10881170

[31] Billadeau DD Nolz JC Gomez TS . Regulation of T-cell activation by the cytoskeleton. Nat Rev Immunol (2007) 7(2):131–43. doi: 10.1038/nri2021 17259969

[32] Powers S Pollack RE . Inducing stable reversion to achieve cancer control. Nat Rev Cancer (2016) 16(4):266–70. doi: 10.1038/nrc.2016.12 27458638

[33] Garrido F Aptsiauri N Doorduijn EM Garcia Lora AM van Hall T . The urgent need to recover mhc class I in cancers for effective immunotherapy. Curr Opin Immunol (2016) 39:44–51. doi: 10.1016/j.coi.2015.12.007 26796069 PMC5138279

[34] Wang S He Z Wang X Li H Liu XS . Antigen presentation and tumor immunogenicity in cancer immunotherapy response prediction. Elife (2019) 8. doi: 10.7554/eLife.49020 PMC6879305 31767055

[35] Brea EJ Oh CY Manchado E Budhu S Gejman RS Mo G . Kinase regulation of human mhc class I molecule expression on cancer cells. Cancer Immunol Res (2016) 4(11):936–47. doi: 10.1158/2326-6066.CIR-16-0177 PMC5110210 27680026

[36] Mizuno S Yamaguchi R Hasegawa T Hayashi S Fujita M Zhang F . Immunogenomic pan-cancer landscape reveals immune escape mechanisms and immunoediting histories. Sci Rep (2021) 11(1):15713. doi: 10.1038/s41598-021-95287-x 34344966 PMC8333422

[37] Chockley PJ Keshamouni VG . Immunological consequences of epithelial-mesenchymal transition in tumor progression. J Immunol (2016) 197(3):691–8. doi: 10.4049/jimmunol.1600458 PMC4955875 27431984

[38] Suarez-Carmona M Lesage J Cataldo D Gilles C . Emt and inflammation: Inseparable actors of cancer progression. Mol Oncol (2017) 11(7):805–23. doi: 10.1002/1878-0261.12095 PMC5496491 28599100

[39] Simon S Labarriere N . Pd-1 expression on tumor-specific T cells: Friend or foe for immunotherapy? Oncoimmunology (2017) 7(1):e1364828. doi: 10.1080/2162402X.2017.1364828 29296515 PMC5739549

[40] Sharpe AH Pauken KE . The diverse functions of the Pd1 inhibitory pathway. Nat Rev Immunol (2018) 18(3):153–67. doi: 10.1038/nri.2017.108 28990585

[41] Khor B Gagnon JD Goel G Roche MI Conway KL Tran K . The kinase Dyrk1a reciprocally regulates the differentiation of Th17 and regulatory T cells. Elife (2015) 4. doi: 10.7554/eLife.05920 PMC4441007 25998054

