# Supplementary figures and images for: The β-carboline Harmine improves the therapeutic benefit of anti-PD1 in melanoma by increasing the MHC-I-dependent antigen presentation
Source: Front Immunol. 2022 Nov 15;13:980704. doi: 10.3389/fimmu.2022.980704 (PMC9705972; doi:10.3389/fimmu.2022.980704)

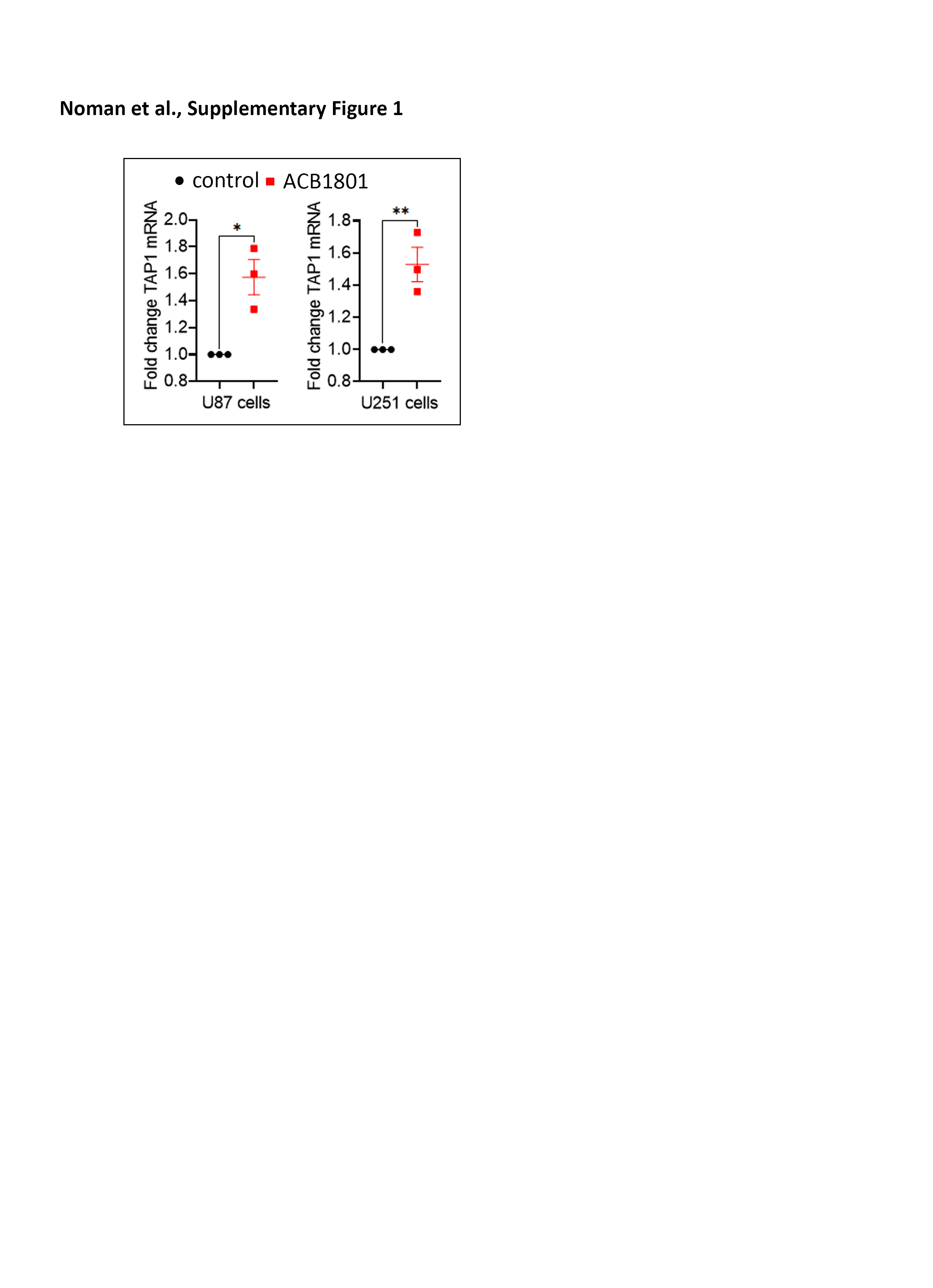

Supplement: Supplementary file 1 [file Image_1.jpeg]

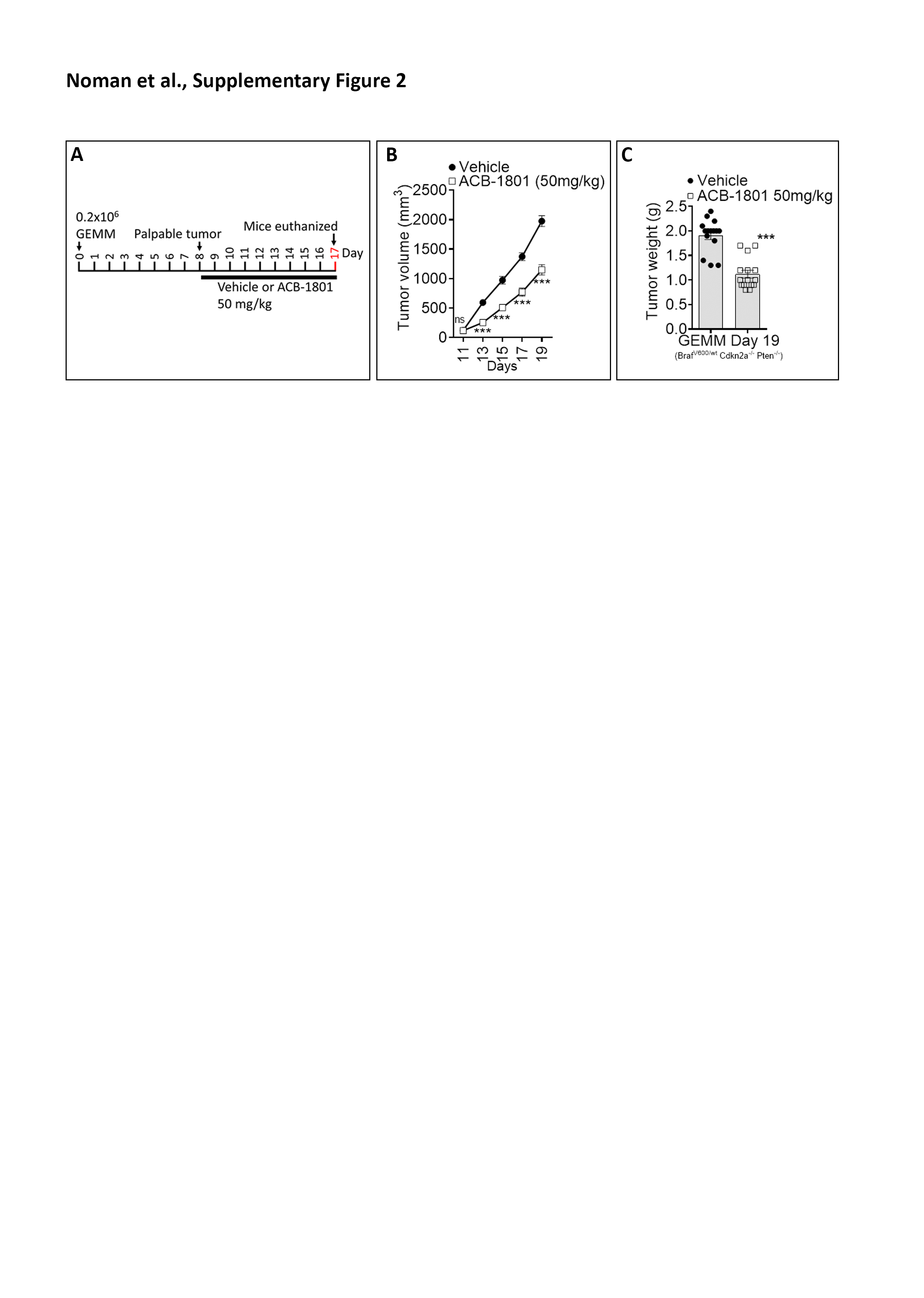

Supplement: Supplementary file 2 [file Image_2.jpeg]

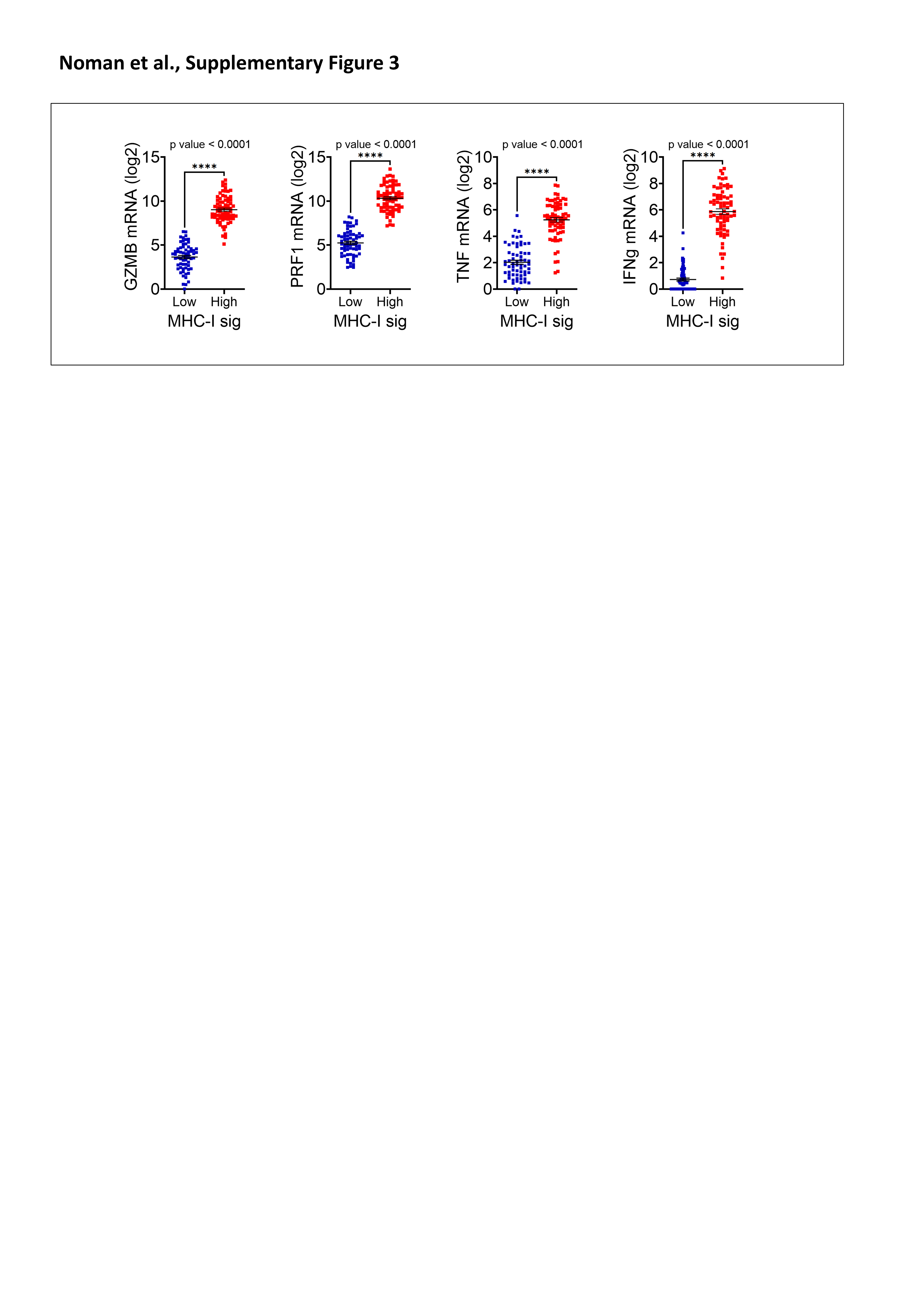

Supplement: Supplementary file 3 [file Image_3.jpeg]

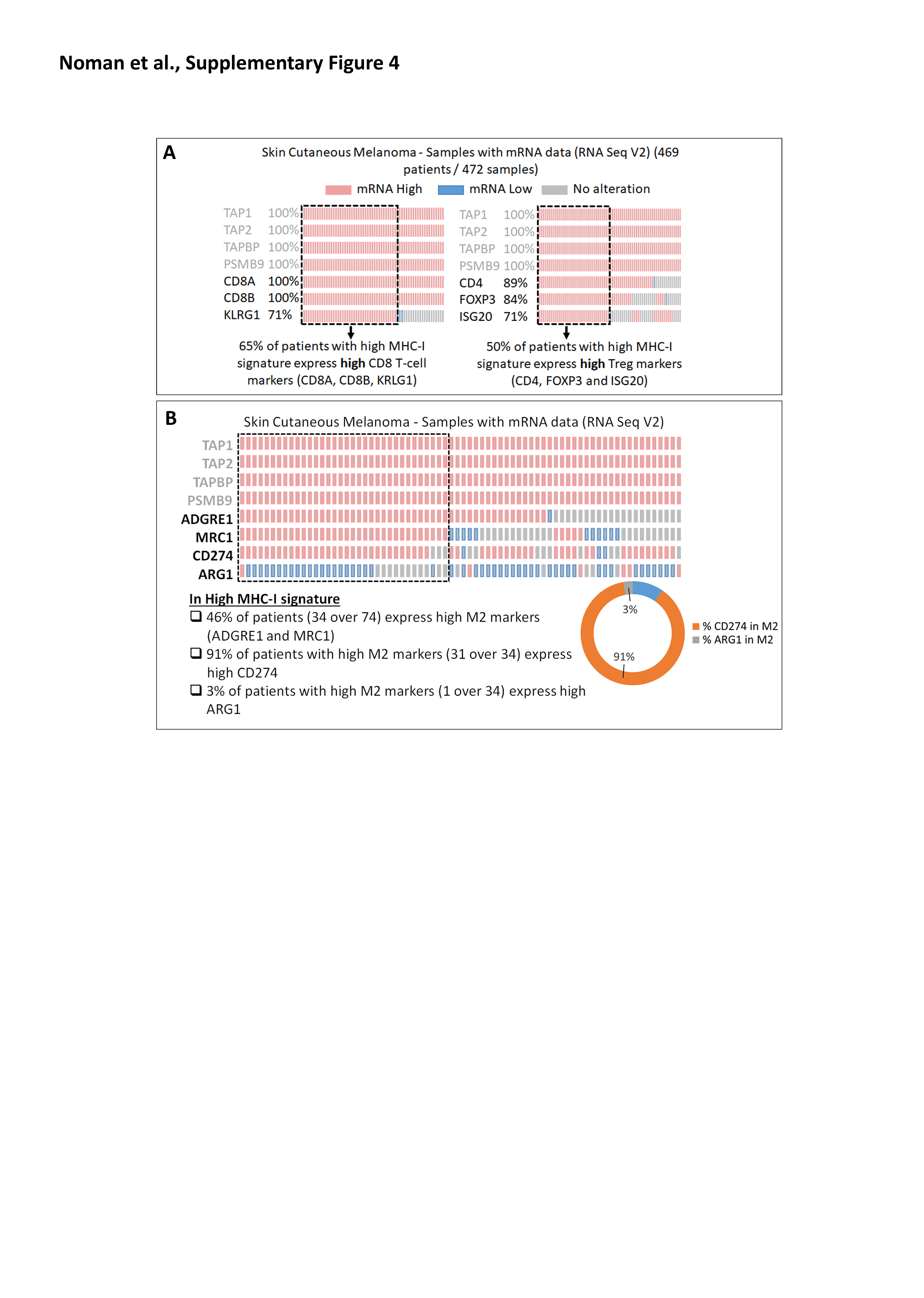

Supplement: Supplementary file 4 [file Image_4.jpeg]
