# Supplementary material for: The β-carboline Harmine improves the therapeutic benefit of anti-PD1 in melanoma by increasing the MHC-I-dependent antigen presentation
Source: Front Immunol. 2022 Nov 15;13:980704. doi: 10.3389/fimmu.2022.980704 (PMC9705972; doi:10.3389/fimmu.2022.980704)
Supplement: Supplementary file 5 [file DataSheet_1.docx]

**Legends for supplementary figures and tables**

**Supplementary Figure 1:**

The mRNA expression of TAP1 in TAP1 in glioblastoma U87 and U251 cells. Cells were treated with 10 uM of ACB-1801 for 24 h. Results are reported as fold change (FC) relative to control cells treated with medium (black bars). Results represent the averages of three independent experiments and are shown as mean ± SEM (error bars). Statistically significant differences low TAP1 were calculated relative to control conditions using an unpaired two-tailed student’s t-test (* =p< 0.05, and ** =p< 0.005).

**Supplementary Figure 2:**

**A:** Experimental schedule of GEMM melanoma treatment with ACB1801. GEMM cells (0.2 x 10^6^ cells) were injected subcutaneously in the right flank of C57BL/6 mice at day 0. Palpable tumors were observed at day 8. Treatment with ACB1801 (50 mg/kg) or vehicle was started at day 8 to day 17 and delivered daily per os. Mice were euthanized at day 17. **B and C:** Tumor growth curves (B) and weight (g) at day 17 (C) of GEMM melanoma in mice treated with vehicle or ACB1801. Results are reported as the average of 15 mice per group as mean ± SEM (error bars). Statistically significant differences low TAP1 are calculated using an unpaired two-tailed student’s t-test (ns= not significant, and ***=p<0.0005).

**Supplementary Figure 3:**

The mRNA expression of cytotoxic markers GZMB, PRF1, TNF and IFNg for NK and CD8 T cells in melanoma patients from TCGA expressing low or high levels of MHC-I signature (MHC-I sig). Data from the TCGA skin cutaneous melanoma (SKCM) cohort (448 patients) were downloaded from cBioPortal (http://www.cbioportal.org/). IDs of patients displaying high and low mRNA expression of MHC-I sig (z-score relative to all samples) were extracted. In patients expressing high and low MHC-I sig, the log2 mRNA expression level (batch normalized from Illumina HiSeq_RNASeqV2) of the indicated cytotoxic markers was identified. The differential expression of genes of interest was found using GraphPad software. Results are represented as the mean ± standard error of the mean (SEM). Statistically significant differences are calculated using an unpaired two-tailed student’s t-test (**** = p<0.0001).

**Supplementary Figure 4:**

**A:** The expression of effector CD8 T-cell markers (CD8A^+^ CD8B^+^ KLRG1^+^) and Treg markers (CD4^+^ Foxp3^+^ ISG20^+^) in melanoma patients displaying high levels of MHC-I signature (TAP1, TAP2, TAPBP, PSMB2). Doted boxes delineate patients expressing high effector CD8 T-cell markers (left) and high Treg markers (right). **B:** The expression of the immunosuppressive markers CD274+ and ARG1+ in MHC-I high melanoma patient displaying high levels of M2 macrophage markers (defined as ADGRE1^+^ MRC1^+^). Doted boxes delineate patients expressing high MHC-I and M2 markers. The doughnut chart represents the percentage of patients displaying high CD274 and ARG1 in M2 macrophages.

**Supplementary tables 1 to 6:**

Information about the TCGA melanoma patients shown in Figure 4. **Table 1** shows the TCGA sample and patient IDs as well as the mRNA expression of MHC-I signature (TAP1, TAP2, TAPBM and PSMB9). **Tables 2 and 3** show the time in months of the overall survival (2) and disease specific survival (3), as well as the status of patients with high and low MHC-I signature (TAP1, TAP2, TAPBM and PSMB9). **Table 4** shows the expression of markers for NK (NCR1 and NCR3) and CD8 (CD8A and CD8B) in patients with high and low MHC-I signature (TAP1, TAP2, TAPBM and PSMB9). **Table 5** shows the expression of cytotoxic markers GZMB, PRF1, TNF and IFNg in patients with high and low MHC-I signature (TAP1, TAP2, TAPBM and PSMB9). **Table 6** shows the expression of inflammatory chemokines (CCL2, CCL4, CCL5, CCL19, CCL21, CXCL9, CXCL10, CXCL11, CXCL13, XCL2) in patients with high and low levels of MHC-I signature (TAP1, TAP2, TAPBM and PSMB9), NK markers and CD8 markers.
